# A Ruptured Pseudoaneurysm of Pancreaticoduodenal Artery: A Case Report

**DOI:** 10.1155/cris/9252686

**Published:** 2026-01-02

**Authors:** Fatemeh Zarimeidani, Ali Saberi, Reza Taheri, Mahtab Sami, Sepideh Soleymani, Mohammadmahdi Dehghan Niri, Rahem Rahmati, Erfan Soleymani, Mohsen Khaleghian, Bahare Hesamifard, Milad Sarafi

**Affiliations:** ^1^ Students Research Committee, Shahrekord University of Medical Sciences, Shahrekord, Iran, skums.ac.ir; ^2^ Department of Surgery, Surgery Research Center, School of Medicine, Rasool-E Akram Hospital, Iran University of Medical Sciences, Tehran, Iran, iums.ac.ir

**Keywords:** case report, false aneurysm, hemorrhage, vascular surgical procedures, visceral artery pseudoaneurysm

## Abstract

**Introduction:**

Pancreaticoduodenal artery (PDA) pseudoaneurysm is a rare occurrence. The intricate clinical manifestations and frequent rupture present challenges in diagnosing and treating the condition.

**Case Presentation:**

A 56‐year‐old man was admitted to the emergency department (ED) with sudden, severe abdominal pain, dizziness, and a history of two fainting events on the same day at home. A decreased blood pressure of 75/60 mmHg was detected on arrival. He had a medical background characterized by a history of gastritis and peptic ulcer disease (PUD) with ongoing use of pantoprazole and sucralfate. A contrast‐enhanced computed tomography (CT) scan revealed retroperitoneal hematoma and a saccular outpouching in the superior and inferior pancreaticoduodenal arcades, in favor of visceral aneurysm, probably with the origin of the PDA. The patient underwent a laparotomy. The ruptured pseudoaneurysm was ligated using 4.0 polypropylene threads, and a Jackson–Pratt drain was inserted. The patient’s recovery following the surgery was uneventful, and he was discharged after 5 days without any issues.

**Conclusion:**

This case highlights the importance of considering a ruptured PDA pseudoaneurysm, which should be evaluated in the differential diagnosis of abdominal discomfort and related symptoms, especially in patients with duodenal ulcers. The successful management of the condition is accomplished through suture ligation, and the diagnosis is effectively made through CT angiography.

## 1. Introduction

Pancreaticoduodenal artery (PDA) pseudoaneurysm, a visceral artery pseudoaneurysm type, is rare. Aneurysms or pseudoaneurysms in the superior and inferior pancreaticoduodenal arteries occur at a rate of about 10% [1]. Pseudoaneurysms resulting from inflammation, trauma, or infection constitute the majority of peripancreatic aneurysms [2]. PDA pseudoaneurysms are critical due to the potential challenges associated with diagnosis and the life‐threatening consequences that can ensue in the event of a rupture. Here, we discuss a rare case of a middle‐aged man with a ruptured pseudoaneurysm of PDA.

## 2. Case Presentation

A 56‐year‐old man was admitted to the emergency department (ED) due to sudden, severe abdominal pain and dizziness associated with two fainting events on the same day at home. On arrival, his vital signs were: temperature 37.1°C, blood pressure 75/60 mmHg, heart rate 123 beats/min, respiratory rate 14 breaths/min, and oxygen saturation 94%. After receiving two consecutive boluses of 1‐L isotonic crystalloid solution (normal saline), his blood pressure stabilized at 121/82 mmHg.

He had a medical background characterized by a history of gastritis and peptic ulcer disease (PUD, duodenal ulcer) with ongoing use of pantoprazole and sucralfate. He also claimed a 10‐year history of abdominal pain, which got worse once he used a squat toilet in the morning. The pain was intense and persistent, with notable exacerbation in the epigastric region. It had radiation to the thoracolumbar. He denied any specific surgical or family history and use of any opioids. Our patient did not have any record of previous pancreatitis. On examination, the patient was alert and oriented. Neurovascular status and skin inspection were normal. Abdominal examination revealed only mild epigastric tenderness, with no guarding or rebound.

**Figure 1:**
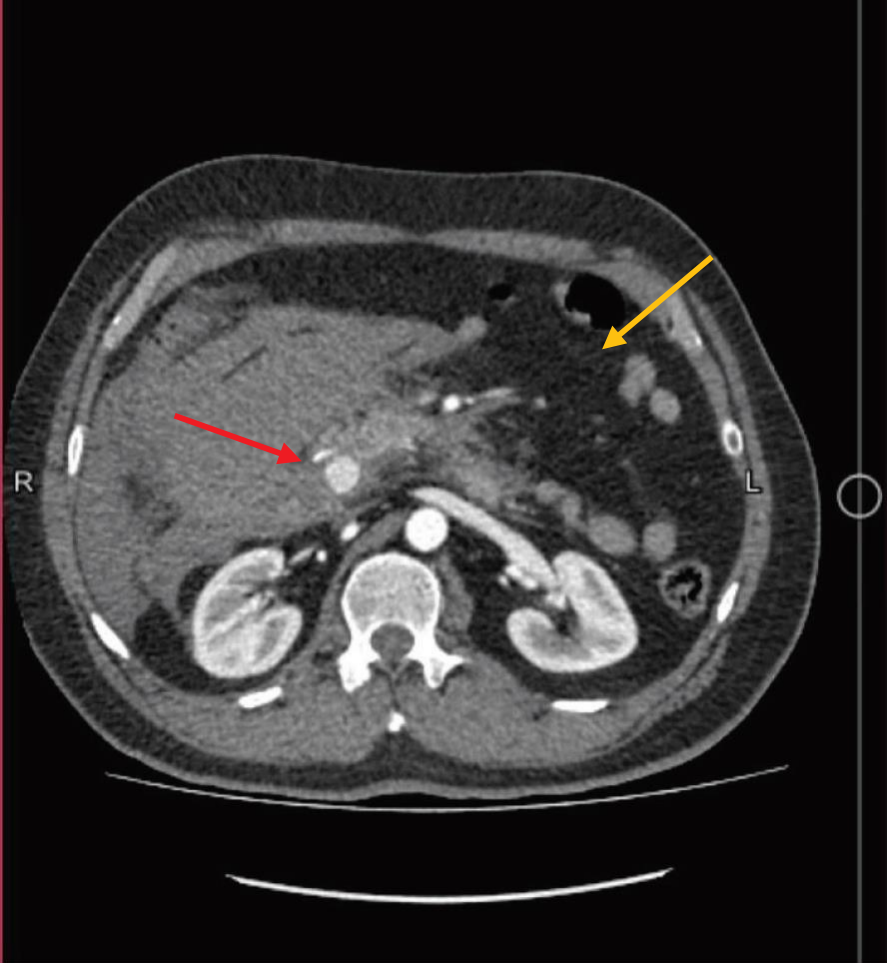
IV contrast‐enhanced CT angiography showing enlarged iso‐to‐hyperdense lesions with significant inflammatory changes around the duodenum, peripancreatic, gastroduodenal, proximal pararenal, and right lateroconal in favor of a large hematoma with a reactive diameter increase of the duodenum and ascending colon (yellow arrow). It also shows a saccular outpouching image consisting of contrast with an irregular wall in the peripancreatic zone in superior and inferior pancreaticoduodenal anastomosis in favor of visceral aneurysm, probably with the origin of the inferior pancreaticoduodenal artery (red arrow). CT, computed tomography.

His laboratory test results on arrival and 6 h later are shown in Tables 1 and 2. Chest X‐ray and ECG were normal. A computed tomography (CT) scan with intravenous contrast (Figure 1) showed enlarged iso‐to‐hyperdense lesions with significant inflammatory changes around the duodenum, peripancreatic, gastroduodenal, proximal pararenal, and right lateroconal in favor of a large hematoma with a reactive diameter increase of the duodenum and ascending colon. It also showed a saccular outpouching image consisting of contrast with an irregular wall in the peripancreatic zone, in superior and inferior pancreaticoduodenal arcades, in favor of visceral aneurysm, probably with the origin of the inferior PDA. He was transferred to the operating room due to the suspicion of bleeding in the laboratory tests and a visceral aneurysm in the imaging.

**Table 1:** Laboratory test results at admission.

Test	Reference range, adults	On admission
Hematocrit (%)	39–52	39.4
Hemoglobin (g/dL)	**14–18**	**12.8**
White‐cell count (per mm^3^)	4000–10,000	9400
Platelet count (per mm^3^)	140,000–440,000	235000
Mean corpuscular volume (fL)	77–97	87.6
Partial‐thromboplastin time, activated (s)	**25–40**	**41**
Prothrombin time (s)	**12–13**	**16.1**
INR (index)	**1–1.1**	**1.27**
Blood urea nitrogen (mg/dL)	5–23	15
Creatinine (mg/dL)	0.5–1.5	1.5
Aspartate aminotransferase (IU/L)	**5–40**	**170**
Alanine aminotransferase (IU/L)	**5–40**	**136**
Creatine phosphokinase (IU/L)	24–195	101
Creatine phosphokinase MB isoenzyme (U/L)	0–24	20
Alkaline phosphatase (IU/L)	64–306	110
Amylase (U/L)	20–104	26
Lactate dehydrogenase (U/L)	**225–500**	**544**
Sodium (mmol/L)	136–145	135
Potassium (mmol/L)	3.7–5.5	4.1
Venous blood pH	7.31–7.41	7.383
Venous blood PCO_2_ (mm Hg)	**41–51**	**32.3**
Venous blood PO_2_ (mm Hg)	35–40	39.3
Venous blood HCO_3_ (meq/L)	**22–26**	**18.8**
Glucose (mg/dL)	**70–115**	**199**
Troponin (high sense‐V) (ng/L)	<19 Normal 19–100 observation >100 massive cardiac damage	4.5
D‐dimer	0–0.6: normal 0.6–1: borderline 1 <: abnormal	0.4

*Note:* To convert the values for glucose to millimoles per liter, multiply by 005551. To convert the values for urea nitrogen to millimoles per liter, multiply by 01357. To convert the values for creatinine to micromoles per liter, multiply by 88.4. To convert the values for bilirubin to micromoles per liter, multiply by 17.1. Reference values are affected by many variables, including the patient population and the laboratory methods used. The ranges used at the Hazrat‐E‐Rasoul General Hospital are for adults who are not pregnant and do not have medical conditions that could affect the results. Therefore, they may not be appropriate for all patients. Abnormal values are emphasized in bold.

**Table 2:** Laboratory test results 6 h after arrival.

*Test*	Reference range, adults	After 6 h
Hematocrit (%)	**39–52**	**34**
Hemoglobin (g/dL)	**14–18**	**10.8**
White‐cell count (per mm^3^)	4000–10,000	6700
Platelet count (per mm^3^)	140,000–440,000	200,000
Mean corpuscular volume (fL)	77–97	87.6
Alkaline phosphatase (IU/L)	64–306	111
Amylase (U/L)	20–104	26
Lactate dehydrogenase (U/L)	**225–500**	**675**
Sodium (mmol/L)	**136–145**	**135**
Potassium (mmol/L)	3.7–5.5	4.1
Venous blood pH	**7.31–7.41**	**7.44**
Venous blood PCO_2_ (mm Hg)	**4151**	**27.5**
Venous blood PO_2_ (mm Hg)	**35–40**	**59.5**
Venous blood HCO_3_ (meq/L)	**22–26**	**18.4**

*Note:* To convert the values for glucose to millimoles per liter, multiply by 005551. To convert the values for urea nitrogen to millimoles per liter, multiply by 01357. To convert the values for creatinine to micromoles per liter, multiply by 88.4. To convert the values for bilirubin to micromoles per liter, multiply by 17.1. Reference values are affected by many variables, including the patient population and the laboratory methods used. The ranges used at the Hazrat‐E‐Rasoul General Hospital are for adults who are not pregnant and do not have medical conditions that could affect the results. Therefore, they may not be appropriate for all patients. Abnormal values are emphasized in bold.

Due to the aneurysm being located in a branch of the PDA with difficult endovascular access and the presence of a large retroperitoneal hematoma compressing adjacent structures, the patient was not considered a good candidate for angiographic embolization. Additionally, the urgency of the situation and the patient’s evolving hemodynamic status necessitated prompt surgical intervention. Therefore, an emergency exploratory laparotomy was performed.

**Figure 3:**
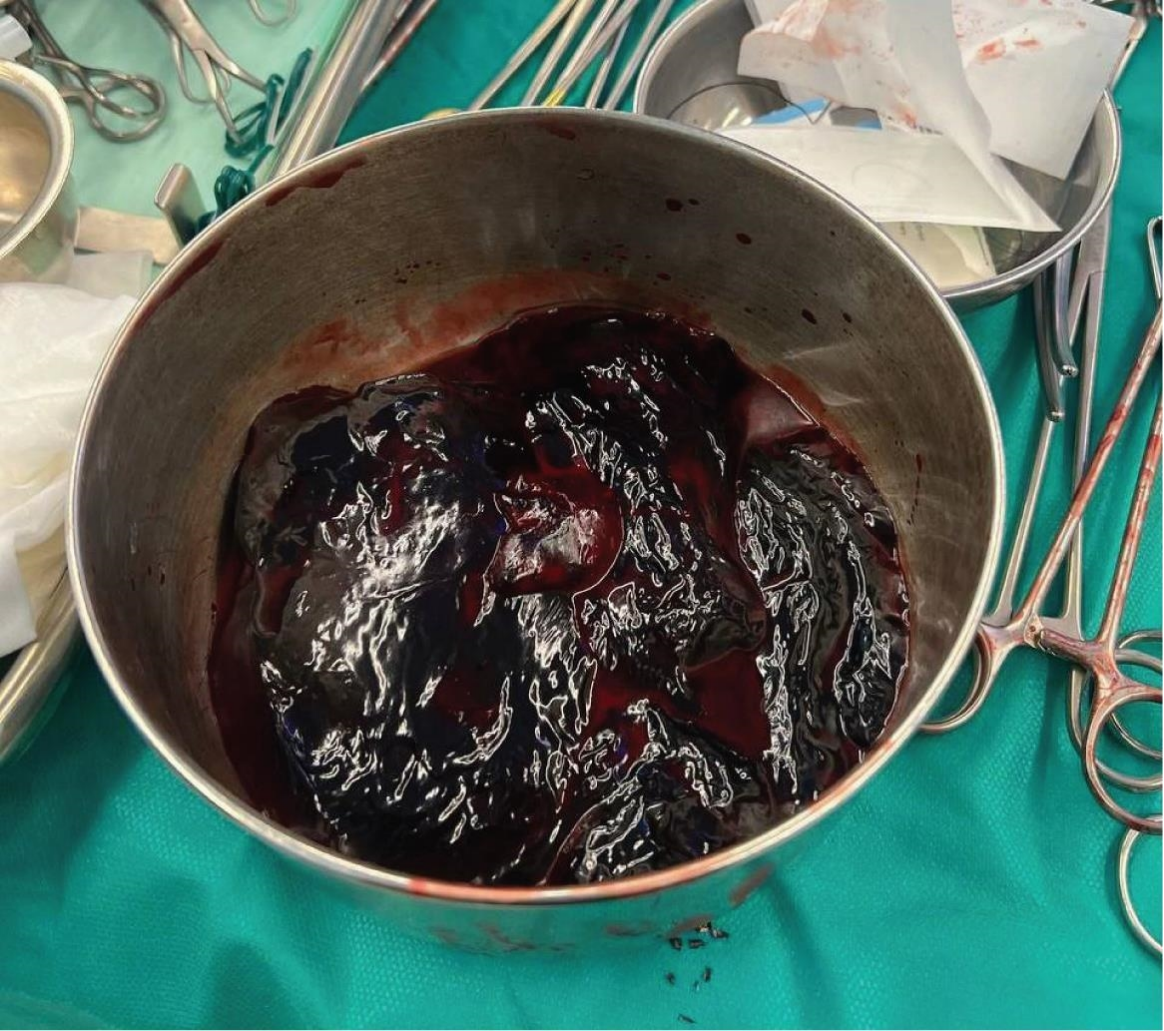
The clots and the hematoma removed from the retroperitoneum.

During the surgical procedure, a midline incision was made. There was no evidence of PUD perforation, pus, fibrin, or fluid in the peritoneum. Approximately 300 cc of blood was also suctioned from the peritoneal cavity. In the retroperitoneum, the hematoma had pushed the C‐loop of the duodenum (Figure 2). Dissection was carried out to expose the abdominal aorta starting from the diaphragmatic level. Once identified, proximal control was achieved using a vascular Satinsky clamp applied to the infrarenal segment of the abdominal aorta. Then, the duodenum was Kocherized, and the parenchyma of the pancreas and the posterior region of the duodenum were explored. Clots and the hematoma were removed (Figure 3), and the ruptured pseudoaneurysm was seen. The bleeding was stopped with the ligation of the superior PDA and inferior PDA with 4.0 polypropylene threads (Figure 2). The location of the aneurysm rupture was packed with 4 × 4 sterile surgical sponges to ensure bleeding control, and the packing was removed after three to 4 min once bleeding had ceased. Due to the rupture being in the area where the head of the pancreas is, a Jackson–Pratt drain was inserted and fixed to the skin by nylon suture to ensure no injury was made to the head of the pancreas and the common bile duct. The fascia was closed by a loop thread. During the surgery, 300 cc of blood was suctioned from the abdomen, and he received two units of packed RBC. The patient’s recovery following the surgery was uneventful (Figure 4), and he was discharged after 5 days without any issues.

Figure 2(A) The massive hematoma splitting the head of the pancreas and the C‐loop of the duodenum. (B) Ligated IPDA and SPDA that caused the massive hematoma. IPDA, inferior pancreaticoduodenal artery; SPDA, superior pancreaticoduodenal artery.

**Figure:**
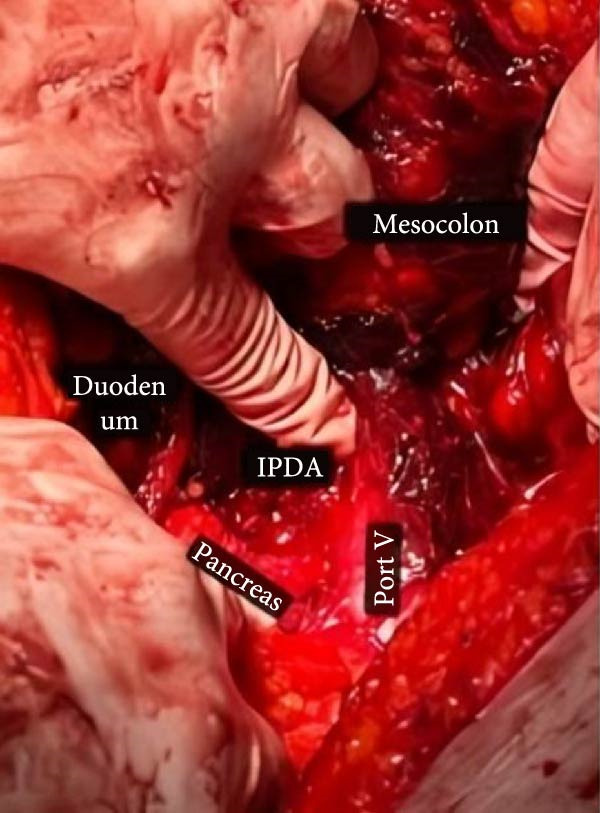
(A)

**Figure:**
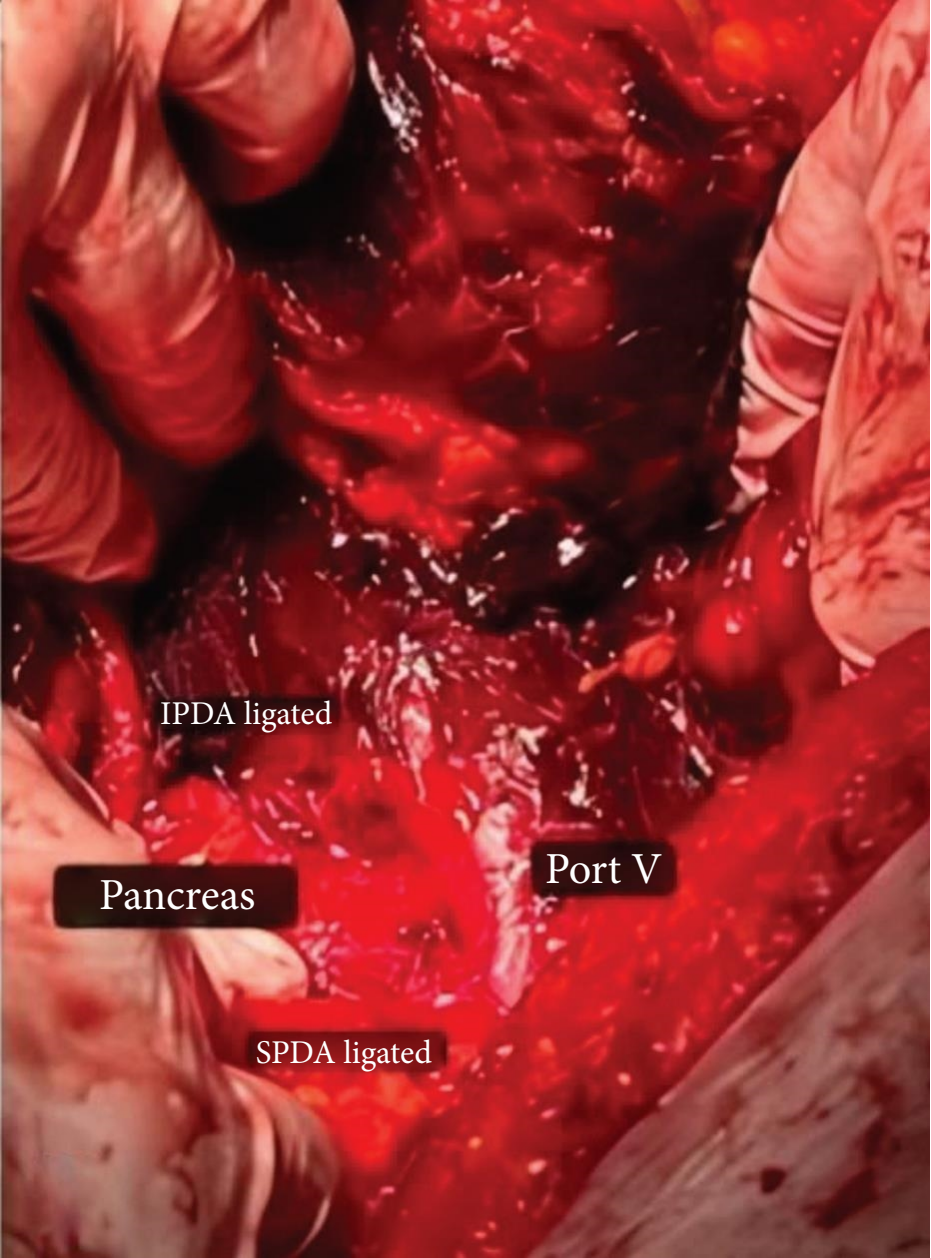
(B)

Figure 4Postoperation IV contrast‐enhanced CT‐angiography. (A) Gastroduodenal artery and (B) celiac artery.

**Figure:**
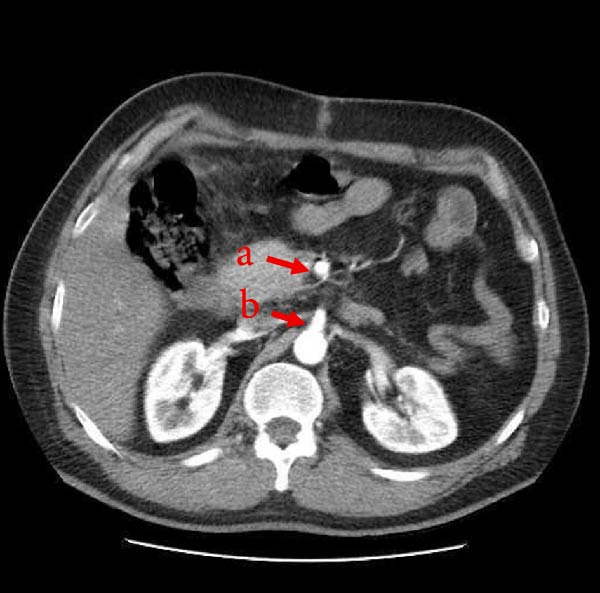
(A)

**Figure:**
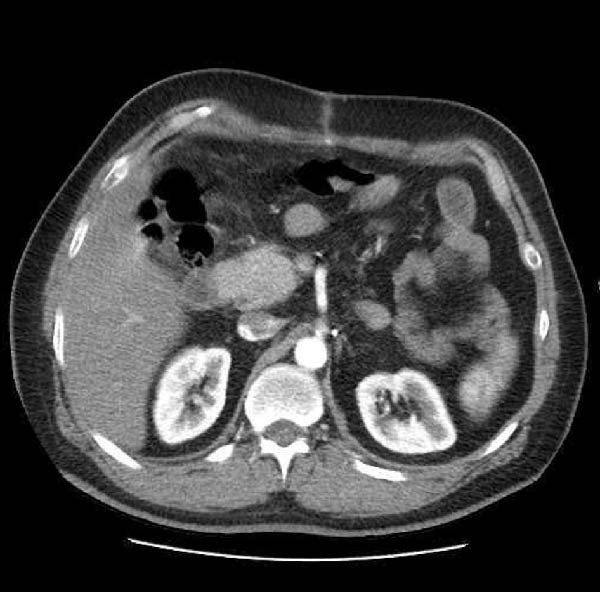
(B)

## 3. Discussion

Approximately 5% of all visceral artery pseudoaneurysms are gastroduodenal or pancreaticoduodenal arcade pseudoaneurysms, which are extremely uncommon [3]. Visceral artery pseudoaneurysms may be asymptomatic or frequently present with nausea/vomiting, a palpable pulsating mass, and, as observed in our case, abdominal pain and hemorrhage symptoms [2]. PDA pseudoaneurysm can develop due to prolonged inflammation in conditions such as PUD, pancreatitis, and malignancies. Additional causes comprise abdominal trauma, such as surgical trauma or Whipple surgery, septic emboli, and iatrogenic injuries [4, 5]. Identifying PDA pseudoaneurysms promptly is crucial due to their rarity and potential to lead to severe consequences.

A pseudoaneurysm is distinguished from a true aneurysm by the absence of arterial components in its wall, which comprises fibrous tissue. This typically leads to continuous enlargement and the formation of a pulsating hematoma [6]. The rupture of PDA pseudoaneurysms is a life‐threatening occurrence with a mortality rate above 25% [5]. This could lead to bleeding in the gastrointestinal system, peritoneal, or retroperitoneal cavity. Our patient manifested retroperitoneal space hemorrhage as acute abdominal pain, hypotension, and anemia. Other presentations of PDA rupture include jaundice, recurrent hematemesis, hematochezia, melena, hemosuccus pancreaticus (bleeding in the pancreatic duct), and hemobilia (bleeding in the bile duct) [6].

The pancreaticoduodenal arcade consists of the gastroduodenal artery, its superior pancreaticoduodenal branches and the inferior pancreaticoduodenal branches of the superior mesenteric artery [2]. Unlike pseudoaneurysms that occur proximally in the gastroduodenal artery, those of the more distal branches of the arcade are often diagnosed postrupture; however, the bleeding is usually confined to the retroperitoneum and is less fatal [3]. In support of this finding, our case had a pseudoaneurysm in the superior and inferior pancreaticoduodenal arcades with adjacency to the inferior PDA, diagnosed after rupturing with a hemorrhage in the retroperitoneum, and was successfully managed with the emergency laparotomy.

Diagnostic modalities of the PDA pseudoaneurysms are ultrasound, CT, angiography, magnetic resonance, or CT angiography. Angiography is the gold standard for evaluating the blood supply of pseudoaneurysms and is a crucial therapy option for stable patients [6]. Contrast‐enhanced multidetector CT angiography is a quick and noninvasive method used as the first‐line diagnostic tool to locate the source and measure the size of pseudoaneurysms, particularly in cases of acute arterial bleeding like our patient [7]. Although ultrasound is a convenient and accessible imaging technique, it cannot identify retroperitoneal hemorrhage [8]. On the other hand, if the patients were allergic to the contrast or had renal failure, magnetic resonance angiography should be considered despite the cost and time [7].

The current standard of pancreatic pseudoaneurysm involves managing bleeding using endovascular embolization or the implantation of a stent [6]. However, hemodynamic status is crucial in determining the visceral pseudoaneurysm treatment approach. Surgery is indicated for patients experiencing hemodynamic instability, when embolization has failed, and in cases of recurring pseudoaneurysm following successful embolization [3, 9]. Our patient was hypotensive with a decrease in his hemoglobin and hematocrit; therefore, we performed an emergency laparotomy. Surgical treatment options for pseudoaneurysms involve removing the affected area and restoring blood flow through revascularization, or ligating with or without removing the surrounding organ [3]. In accordance, we successfully conducted a suture ligation. Overall, surgical approaches can be complex and are linked with higher postoperative complications and mortality, making endovascular methods more appealing when feasible [9].

## 4. Conclusion

Pseudoaneurysm of PDA is a rare entity with a high potential for rupture, resulting in hemodynamic instability. It is imperative to identify and address this complication to avoid catastrophic consequences promptly. Herein, we reported a rare occurrence of a ruptured pancreaticoduodenal pseudoaneurysm in a patient with a previous duodenal ulcer. The patient’s condition was determined by the use of CT angiography and was effectively treated by suture ligation.

## Ethics Statement

Our institution does not require ethical approval for case report research as long as the publication contains any personal information about the patient.

## Consent

The purpose of this study was explained to the patient, and his informed consent was obtained to publish his information and any accompanying photos.

## Conflicts of Interest

The authors declare no conflicts of interest.

## Author Contributions

Conceptualization: Ali Saberi and Reza Taheri. Project administration: Milad Sarafi. Investigation, resources, visualization: Mahtab Sami, Sepideh Soleymani, Mohammadmahdi Dehghan Niri, and Erfan Soleymani. Supervision and validation: Ali Saberi, Reza Taheri, Mohsen Khaleghian, and Bahare Hesamifard. Writing – original draft: Fatemeh Zarimeidani and Rahem Rahmati. Writing – review and editing: Fatemeh Zarimeidani, Rahem Rahmati, and Milad Sarafi.

## Funding

This report did not receive any financial assistance.

## Data Availability

The data that support the findings of this study are available from the corresponding author upon reasonable request.
